# Usefulness of Transbronchial Lung Cryobiopsy When Starting Antifibrotic Treatment and Predicting Progressive Fibrosing Interstitial Lung Disease: Descriptive Research

**DOI:** 10.1111/crj.13809

**Published:** 2024-07-16

**Authors:** Makiko Takatsuka, Hideaki Yamakawa, Tamiko Takemura, Shintaro Sato, Hiroki Ohta, Kenji Kusano, Tomohiro Oba, Rie Kawabe, Keiichi Akasaka, Hiroki Sasaki, Masako Amano, Jun Araya, Hidekazu Matsushima

**Affiliations:** ^1^ Department of Respiratory Medicine Saitama Red Cross Hospital Saitama Japan; ^2^ Department of Respiratory Medicine Tokyo Jikei University Hospital Tokyo Japan; ^3^ Department of Pathology Kanagawa Cardiovascular and Respiratory Center Yokohama Japan; ^4^ Department of Radiology Saitama Red Cross Hospital Saitama Japan

**Keywords:** fibrotic hypersensitivity pneumonitis, idiopathic, interstitial lung disease, therapy, transbronchial lung cryobiopsy

## Abstract

**Background:**

Although transbronchial lung cryobiopsy (TBLC) is widely used in diagnostic algorithms for various interstitial lung diseases (ILDs), its real‐world utility in the therapeutic decision‐making strategy for ILD patients remains unclear, in particular, when judging the time to start antifibrotic agents.

**Methods:**

We analyzed medical records of 40 consecutive patients with idiopathic or fibrotic hypersensitivity pneumonitis who underwent TBLC. A TBLC‐based usual interstitial pneumonia (UIP) score was used to assess three morphologic descriptors: patchy fibrosis, fibroblastic foci, and honeycombing.

**Results:**

In our 40 patients with ILD, the most frequent radiological feature was indeterminate for UIP (45.0%). Final diagnosis included idiopathic pulmonary fibrosis (22.5%), fibrotic nonspecific interstitial pneumonia (5.0%), fibrotic hypersensitivity pneumonitis (35.0%), and unclassifiable ILD (37.5%). Linear mixed‐effects analysis showed that declines in the slopes of %FVC and %DL_CO_ in patients with TBLC‐based UIP “Score ≥ 2” were significantly steeper than those of patients with “Score ≤ 1.” During follow‐up of patients with Score ≥ 2 (*n* = 24), more than half of them (*n* = 17) received an antifibrotic agent, with most patients (*n* = 13) receiving early administration of the antifibrotic agent within 6 months after the TBLC procedure.

**Conclusions:**

TBLC‐based UIP Score ≥ 2 indicated the increased possibility of a progressive fibrosis course that may prove helpful in predicting progressive pulmonary fibrosis/progressive fibrosing ILD even if disease is temporarily stabilized due to anti‐inflammatory agents. Patients may benefit from early introduction of antifibrotic agents by treating clinicians.

## Graphical abstract

Although transbronchial lung cryobiopsy (TBLC) is widely used in diagnostic algorithms for various interstitial lung diseases (ILDs), its real‐world utility in the therapeutic decision‐making strategy for ILD patients remains unclear. In the present study, TBLC‐based UIP Score ≥ 2 indicated the increased possibility of a progressive fibrosis course that may prove helpful in predicting progressive pulmonary fibrosis/progressive fibrosing ILD.

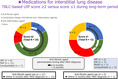

## 1 Introduction

The interstitial lung diseases (ILDs) represent a broad spectrum of conditions that comprise many heterogeneous groups and subgroups, all of which are characterized by inflammation and fibrosis of the alveolar wall with impairment of gas exchange leading to progressive dyspnea and disability [1]. The most common of these conditions is idiopathic pulmonary fibrosis (IPF). In addition, idiopathic fibrotic nonspecific interstitial pneumonia (ifNSIP), fibrotic hypersensitivity pneumonitis (fHP), connective tissue disease‐associated ILD (CTD‐ILD), smoking‐related ILDs (SR‐ILDs), and other uncommon ILDs are known [1]. Notably, ILDs are a diverse group of pulmonary disorders with differing pathogenic mechanisms, treatment options, and disease behaviors [2]. Recently, a clinical trial reported a beneficial effect of an antifibrotic agent in ILDs other than IPF that manifest progressive pulmonary fibrosis (PPF) or progressive fibrosing ILD (PF‐ILD) [3, 4], prompting a paradigm shift toward an en bloc approach to antifibrotic therapy [2]. However, clinicians often raise concern because it is unclear whether standard treatment (i.e., anti‐inflammatory and/or antifibrotic agent) is adequate, in particular, when judging the time to start an antifibrotic agent.

Recently, the usefulness of transbronchial lung cryobiopsy (TBLC) has been recognized, and its use has widened to be included in the diagnostic algorithm of various ILDs [5, 6, 7, 8]. In addition, TBLC may be a good option when determining treatment strategy for ILDs in patients with disease progression [9, 10]. In CTD‐ILD, anti‐inflammatory therapy is generally accepted as the cornerstone of treatment because autoimmune‐mediated pulmonary inflammation is considered a key pathobiological pathway in CTD [11]. In addition, findings vary significantly by ILD subtype, with CTD‐ILD showing little change in FVC after satisfying most PF‐ILD criteria, whereas other ILDs such as idiopathic ILD and fHP show significantly larger decline [12]. In other words, the risk of a progressive fibrotic course requiring antifibrotic therapy is higher in idiopathic ILD and fHP than in CTD‐ILD. Moreover, the poor reported interobserver agreement across experienced multidisciplinary teams for the diagnosis of fHP suggests a lack of agreement on the specific features that define fHP and those that distinguish it from idiopathic fibrosing ILD. Taken together, accurate separation of idiopathic ILD from fHP is crucial to patient management but is frequently a difficult problem [13]. Therefore, the aim of this study was to evaluate whether TBLC can modify the treatment strategy of ILD focusing on idiopathic ILD and fHP, in particular, at the time that antifibrotic treatment is started.

## 2 Materials and Methods

### 2.1 Patients

We selected 59 consecutive patients with ILD who underwent TBLC in Saitama Red Cross Hospital between April 2018 and March 2020. Among them, 16 patients with CTD‐ILD were excluded, as were 3 patients with nonrespiratory‐related death during follow‐up. This study on humans was conducted according to guidelines of the Declaration of Helsinki and approved by the Ethics Committee of Medical Research, Saitama Red Cross Hospital, Japan (approval no. 19‐C, 23 May 2019). Informed consent was waived by the Ethics Committee of Medical Research because of the retrospective nature of this study.

### 2.2 Data Collection

Baseline clinical measures were obtained within 3 months before the TBLC as in our previous study [10]. Each patient's high‐resolution computed tomography (HRCT) scan was reviewed in a multidisciplinary discussion (MDD) and classified as presenting an HRCT pattern of usual interstitial pneumonia (UIP), probable UIP, indeterminate for UIP, or alternative diagnosis according to the American Thoracic Society/European Respiratory Society/Japanese Respiratory Society/Latin American Thoracic Association guideline 2018 [10, 13]. For pulmonary emphysema, positive findings of emphysema were visually defined as the presence of an area of low attenuation indicating the lack of a distinct alveolar wall threshold over 10% [14]. Final diagnosis by MDD included idiopathic disease (IPF, fNSIP, and unclassifiable) and fHP based on previous criteria [2, 15, 16]. The term “unclassifiable ILD” was introduced in the consensus classification to encompass a subset of ILDs that cannot be classified within the confines of our current diagnostic framework. Therefore, unclassifiable (idiopathic) was defined by results from either inadequate or discordant clinical, radiologic, and pathologic data, such that a specific ILD diagnosis was not possible according to previous criteria [2, 15].

### 2.3 Transbronchial Cryobiopsy Procedure

As in our previous study [17], patients were anesthetized with intravenous midazolam and fentanyl, and 2% lidocaine was added intratracheally as appropriate. An endotracheal tube was inserted for airway control. An endobronchial balloon catheter was routinely used for bronchial blockade. A flexible reusable cryoprobe of either 1.9 or 2.4 mm in diameter was inserted from a peripheral bronchus to just below the pleura under fluoroscopy [17]. After freezing the probe for 5–7 s, the bronchoscope was withdrawn along with the biopsy specimen and probe, and the endobronchial balloon catheter was inflated simultaneously [17]. All adverse events were defined according to a previous study [18].

### 2.4 Pathological Evaluation

An expert pathologist (T. Takemura) evaluated TBLC specimens for pathological quality, quantity, and confidence according to previous reports [18, 19]. The quality (Grade A/B/C) and quantity (Level A/B/C) of the tissue specimens were classified into three grades according to a previous study [18].

Major histologic patterns were classified according to the classification of idiopathic interstitial pneumonias published in 2013 and fHP published in 2020 [16, 20]. Characteristic histologic features such as lymphoid aggregates with germinal centers or prominent lympho‐plasmacytic infiltration indicated “prominent inflammatory cells” according to previous reports [10, 21]. In addition, to identify a UIP pattern, a TBLC‐based UIP score was determined according to previously reported criteria by Ravaglia et al. [5] A score of 1, 2, or 3 points was applied based on the presence of one, two, or all three of the following morphologic descriptors: patchy fibrosis, fibroblastic foci (FF), and honeycombing [5, 10]. We defined two subgroups with “Score ≥ 2” and “Score ≤ 1” according to the TBLC‐based UIP score.

### 2.5 Statistical Analysis

Categorical baseline characteristics are summarized by frequency and percentage, and continuous characteristic are reported as the mean ± SD. To detect differences between groups as Score ≥ 2 and Score ≤ 1, Fisher's exact test, unpaired *t* test, or Mann–Whitney *U* test was used as appropriate. If pulmonary function testing was repeatedly performed after the initial assessment, long‐term changes in respiratory function were evaluated. We created a chart of the time data of %forced vital capacity (%FVC) and %diffusing capacity of the lungs for carbon monoxide (%DL_CO_) via a scatter plot for each patient and then added a regression line. The intercept (steepness of the line) and slope (location where the line intersects an axis) of the curves for FVC and %DL_CO_ of each patient were extracted. Analysis of serial changes in the levels of %FVC and %DL_CO_ in the patients with Score ≥ 2 and Score ≤ 1 was performed with linear mixed effects models. We considered *p* < 0.05 to represent statistical significance. All data were analyzed using SPSS version 22.0 (IBM Japan, Tokyo, Japan).

## 3 Results

### 3.1 Patient Characteristics

**FIGURE 1:**
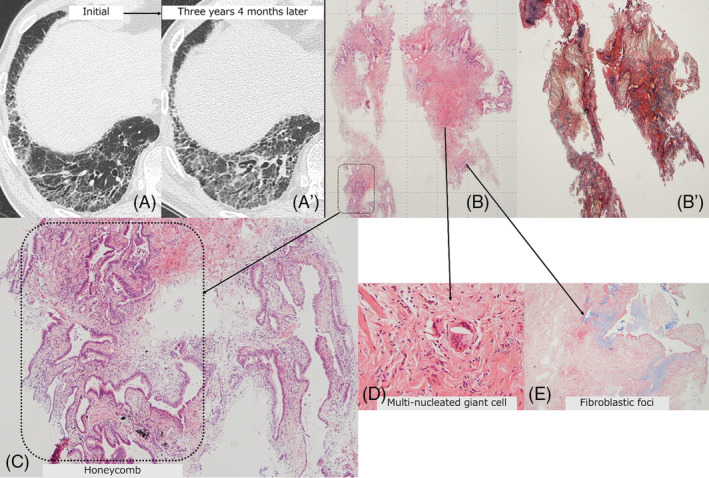
Case 1: TBLC‐based UIP score 2 in a 66‐year‐old man with fibrotic hypersensitivity pneumonitis. (A, A′) Radiological time‐dependent changes showed progressive pulmonary fibrosis after 3 years and 4 months. (B, B′) The TBLC specimens revealed the presence of fibrosis with elastosis located at the perilobular area (B: ×1; hematoxylin and eosin [HE] staining; B′: ×1; Elastica van Gieson staining). (C–E) High‐power magnification view of the dotted rectangle in (B) shows a honeycomb‐like lesion (C) (×4, HE staining), multi‐nucleated giant cell (D) (×20, HE staining), and fibroblastic foci (E) (×20, Alcian blue staining).

**FIGURE 2:**
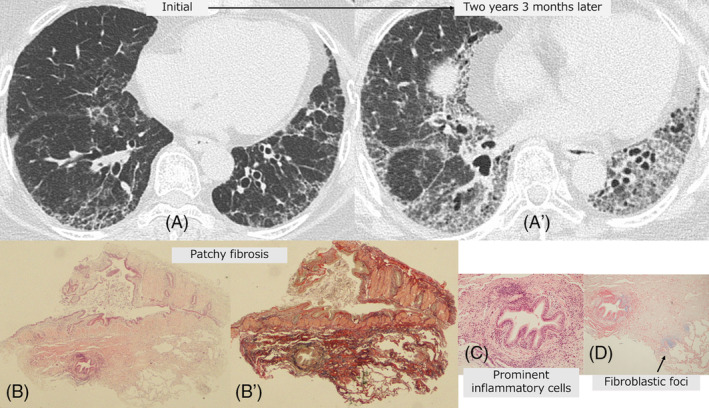
Case 2: TBLC‐based UIP score 2 in a 65‐year‐old woman with fibrotic hypersensitivity pneumonitis. (A, A′) Radiological time‐dependent changes showed progressive pulmonary fibrosis after 2 years and 3 months. (B, B′) The TBLC specimens showed the lesion to be characterized by dense collagenous fibrosis with architectural destruction as patchy fibrosis (B: ×1; hematoxylin and eosin [HE] staining; B′: ×1; Elastica van Gieson staining). (C, D) High‐power magnification view shows prominent lympho‐plasmacytic inflammation around the bronchiole (C) (×10, HE staining) and fibroblastic foci (arrow) (E) (×4, Alcian blue staining).

**FIGURE 3:**
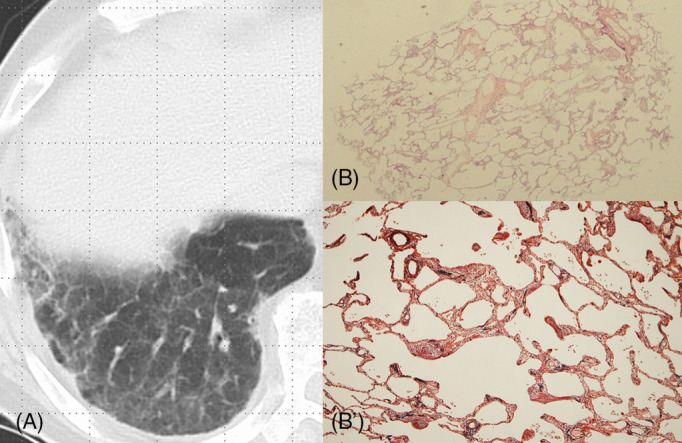
Case 3: TBLC‐based UIP score 0 in a 70‐year‐old man with idiopathic fibrotic nonspecific interstitial pneumonia. (A) Chest radiological image shows ground‐glass opacity with lower lobe distribution. (B, B′) TBLC specimen shows uniform interstitial thickening with mild chronic inflammation (B: ×1; hematoxylin and eosin staining; B′: ×4; Elastica van Gieson staining).

In our cohort of 40 patients with ILD, 25 patients (62.5%) were male, mean age was 69.4 years, and 22 patients (55.0%) had a no history of smoking (Table 1). The most frequent radiological feature in the present study was indeterminate for UIP (*n* = 18, 45.0%), followed by alternative diagnosis (20.0%), probable UIP (30.0%), and UIP (5.0%). Overall pulmonary function test results showed a mean %FVC of 79.9% and %DL_CO_ of 66.7%. The most frequent pathological feature was that of unclassifiable (*n* = 16, 40.0%), followed by UIP (27.5%), HP (25.0%), and NSIP (7.5%) (Table 2). The framework of pathological HP included UIP (80.0%), NSIP (10.0%), and mixed NSIP/UIP (10.0%). Among the pathological findings, patchy fibrosis was present in 27 patients (67.5%), FF in 37 patients (77.5%), honeycombing in 5 patients (12.5%), and prominent inflammatory cells in 9 patients (22.5%). Concrete examples of the findings of patchy fibrosis, FF, honeycombing, and prominent inflammatory cells are shown in Figures 1, 2, 3. The final diagnoses after MDD included 9 patients (22.5%) with IPF, 2 patients (5.0%) with ifNSIP, 14 patients (35.0%) with fHP, and 15 patients (37.5%) with unclassifiable ILD, which included mixed NSIP/UIP (*n* = 12), mixed NSIP/organizing pneumonia (*n* = 1), SR‐ILD (*n* = 1), and prominent inflammatory cells with UIP (*n* = 1).

**TABLE 1:** Patient characteristics (*n* = 40).

Characteristics	Overall value	TBLC‐based UIP score		*p* value
		2 (*n* = 23) and 3 (*n* = 3) (total, *n* = 26)	0 (*n* = 6) and 1 (*n* = 8) (total, *n* = 14)	
Male, *n* (%)	25 (62.5%)	18 (69.2%)	7 (50.0%)	0.310
Age (years), mean ± SD	69.4 ± 7.7	71.4 ± 5.4	65.6 ± 9.8	0.021
Never smoker, *n* (%)	18 (45.0%)	13 (50.0%)	5 (35.7%)	0.510
BMI (kg/m^2^), mean ± SD	25.3 ± 3.3	25.2 ± 3.5	25.5 ± 3.1	0.815
KL‐6 (U/mL), mean ± SD	1525.0 ± 1109.7	1393.8 ± 1200.0	1768.6 ± 909.4	0.314
SP‐D (ng/mL), mean ± SD	436.4 ± 329.2	405.3 ± 324.5	494.3 ± 342.1	0.422
%FVC, mean ± SD	79.9 ± 17.4	80.2 ± 17.2	79.4 ± 18.4	0.901
%DL_CO_, mean ± SD	66.7 ± 23.0	67.6 ± 18.6	65.1 ± 30.3	0.760
BAL				
Lymphocytes (%), mean ± SD	21.4 ± 20.6	19.7 ± 17.9	24.8 ± 25.7	0.489
Neutrophils (%), mean ± SD	7.1 ± 7.4	6.5 ± 7.1	8.3 ± 8.0	0.492
Eosinophils (%), mean ± SD	3.8 ± 5.0	3.1 ± 3.7	5.1 ± 6.8	0.279
Radiological honeycombing	10 (25.0%)	7 (26.9%)	3 (21.4%)	> 0.999
HRCT pattern				
UIP	2 (5.0%)	1 (3.8%)	1 (7.1%)	0.275
Probable UIP	12 (30.0%)	9 (34.6%)	3 (21.4%)	
Indeterminate	18 (45.0%)	13 (50.0%)	5 (35.7%)	
Alternative	8 (20.0%)	3 (11.5%)	5 (35.7%)	

Abbreviations: BAL, bronchoalveolar lavage; BMI, body mass index; DL_CO_, diffusing capacity for the lung carbon monoxide; FVC, forced vital capacity; HRCT, high‐resolution computed tomography; KL‐6, Krebs von den Lungen‐6; SD, standard deviation; SP‐D, surfactant protein‐D; TBLC, transbronchial lung cryobiopsy; UIP, usual interstitial pneumonia.

**TABLE 2:** Pathological findings and outcome (*n* = 40).

Findings	No. of patients (total)	TBLC‐based UIP score		*p* value
		2 (*n* = 23) and 3 (*n* = 3) (total, *n* = 26)	0 (*n* = 6) and 1 (*n* = 8) (total, *n* = 14)	
Pathological pattern				
UIP	11 (27.5%)	10 (38.5%)	1 (7.1%)	0.009
NSIP	3 (7.5%)	0 (0.0%)	3 (21.4%)	
HP	10 (25.0%)	8 (30.8%)	2 (14.3%)	
Unclassifiable	16 (40.0%)	8 (30.8%)	8 (57.1%)	
Pathological findings, *n* (%)				
Patchy fibrosis	27 (67.5%)	24 (92.3%)	3 (21.4%)	<0.001
FF	31 (77.5%)	26 (100.0%)	5 (35.7%)	<0.001
Honeycomb	5 (12.5%)	5 (19.2%)	0 (0.0%)	0.143
Prominent inflammatory cells	9 (22.5%)	3 (11.5%)	6 (42.9%)	0.044
Lymphoid follicle	1 (2.5%)	1 (3.8%)	0 (0.0%)	>0.999
Diagnosis after MDD				
IPF	9 (22.5%)	7 (26.9%)	2 (14.3%)	0.114
NSIP	2 (5.0%)	0 (0.0%)	2 (14.3%)	
fHP	14 (35.0%)	11 (42.3%)	3 (21.4%)	
Unclassifiable (idiopathic)	15 (37.5%)	8 (30.8%)	7 (50.0%)	
Total deaths (during follow‐up), *n* (%)	18 (45.0%)	11 (42.3%)	7 (50.0%)	0.744
Acute exacerbation of ILD	7 (17.5%)	2 (7.7%)	5 (35.7%)	0.077
Chronic disease progression	9 (22.5%)	7 (26.9%)	2 (14.3%)	
Pulmonary infection	2 (11.1%)	2 (18.2%)	0 (0.0%)	
Median follow‐up, months (range)	40 (7–58)	42 (9–58)	36 (7–57)	0.364

Abbreviations: FF, fibroblastic foci; fHP, fibrotic hypersensitivity pneumonitis; HP, hypersensitivity pneumonitis; ILD, interstitial lung disease; IPF, idiopathic pulmonary fibrosis; MDD, multi‐disciplinary discussion; NSIP, nonspecific interstitial pneumonia; TBLC, transbronchial lung cryobiopsy; UIP, usual interstitial pneumonia.

### 3.2 Survival

During the median 40‐month follow‐up period, 18 patients (45.0%) died due to respiratory‐related disease, which included acute exacerbation (*n* = 7), chronic disease progression (*n* = 9), and pulmonary infection (*n* = 2) (Table 2).

### 3.3 Comparison Between TBLC‐Based UIP Score ≥ 2 and Score ≤ 1

**FIGURE 4:**
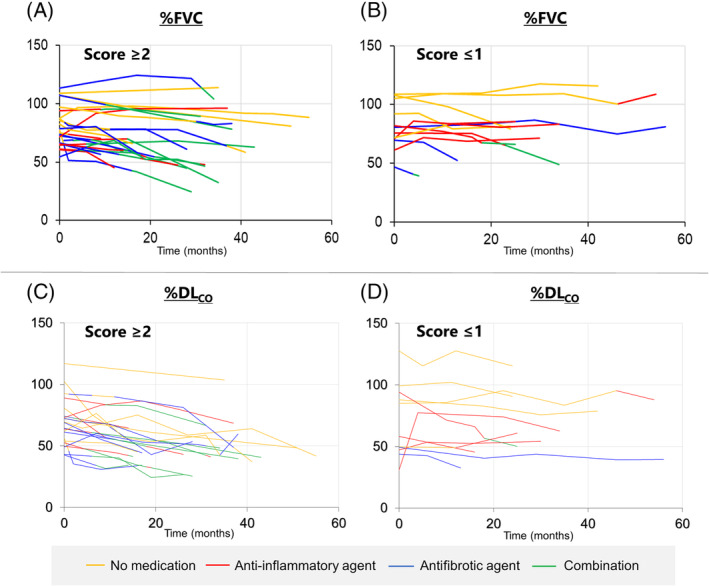
Spaghetti plots of %FVC (*n* = 36) and %DL_CO_ (*n* = 33) for each group are shown along the time axis with the color‐coded therapeutic agents used. (A) %FVC in the patient group with Score ≥ 2; (B) %FVC in the patient group with Score ≤ 1; (C) %DL_CO_ in the patient group with Score ≥ 2; (D) %DL_CO_ in the patient group with Score ≤ 1.

**FIGURE 5:**
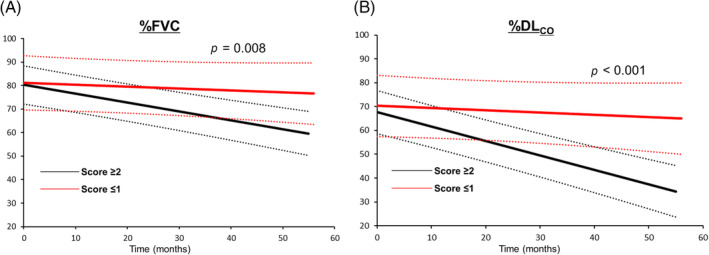
Linear mixed‐effects analysis of changes in FVC (A) and %DL_CO_ (B) during follow‐up. (A) The declining slope of %FVC in patients with Score ≥ 2 (mean, −37.5 mL/month [95% CI: −50.2, −24.8]) was significantly steeper than that of patients with Score ≤ 1 (mean, −8.2 mL/month [95% CI: −25.5, −9.1]) (*p* = 0.008). (B) The declining slope of %DL_CO_ in patients with Score ≥ 2 (mean, −0.60%/month [95% CI: −0.76, −0.45]) was significantly steeper than that in patients with Score ≤ 1 (mean, −0.08%/month [95% CI: −0.26, +0.09]) (*p* < 0.001).

The patients with Score ≥ 2 were older and had a high frequency of pathological UIP pattern (Tables 1 and 2). Factors such as sex, smoking history, pulmonary function, bronchiolar alveolar lavage findings, and radiological pattern were not significantly different between the patients with Score ≥ 2 and Score ≤ 1. Compared with patients with Score ≥ 2, those with Score ≤ 1 had a high frequency of prominent inflammatory cells. In terms of the disease course, spaghetti plots of %FVC (*n* = 36) and %DL_CO_ (*n* = 33) for each group are shown along the time axis in Figure 4. The plots are color‐coded according to the therapeutic agents used. Linear mixed‐effects analysis showed that the declines in the slope of %FVC and %DL_CO_ in the patients with Score ≥ 2 were significantly steeper than those in the patients with Score ≤ 1 (Figure 5).

**FIGURE 6:**
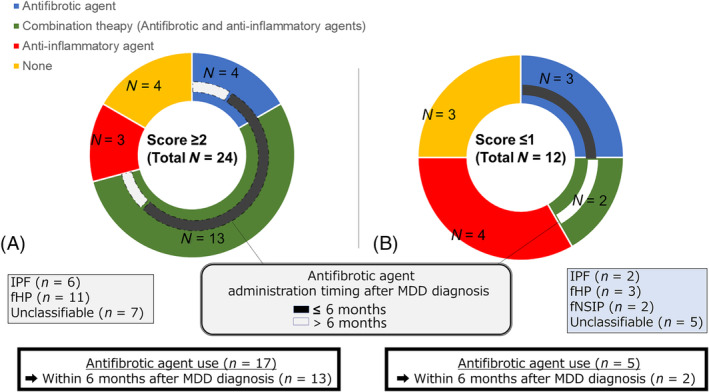
Medications for interstitial lung disease. (A) The patients with TBLC‐based UIP Score ≥ 2. During the follow‐up period of these 24 patients, more than half of them (*n* = 13, 54.2%) received combination therapy. In the groups with antifibrotic agent use (total, *n* = 17), many patients (*n* = 13, 76.5%) received administration of an antifibrotic agent within 6 months after the MDD‐determined diagnosis. Among the groups with combination therapy, most patients (*n* = 11, 64.7%) were administered an antifibrotic agent within 6 months of the MDD‐determined diagnosis. (B) The patients with TBLC‐based UIP Score ≤ 1. In these 12 patients, 5 received antifibrotic agents, among whom 2 patients received combination therapy. The period of antifibrotic agent administration in these 2 patients was begun no earlier than 6 months after the MDD‐determined diagnosis.

Focusing on medications administered for ILD (Figure 6), among the patients with a Score ≥ 2 TBLC‐based UIP score, 6 patients had IPF, 11 had fHP, and 7 had unclassifiable ILD. During the follow‐up period for these 24 patients, more than half of them (*n* = 13, 54.2%) received combination therapy (i.e., anti‐inflammatory and antifibrotic agents). An antifibrotic agent was added to the anti‐inflammatory agent in 4 patients, and an anti‐inflammatory agent was added to the antifibrotic agent in 9 patients. Moreover, many patients (*n* = 13, 76.5%) received antifibrotic agent administration within 6 months after the diagnosis determined by MDD in the groups with antifibrotic agent use (*n* = 17). From the perspective of the group with combination therapy (*n* = 13), most patients (*n* = 11, 84.6%) were administered an antifibrotic agent within 6 months of the MDD‐determined diagnosis.

Contrastingly, patients with a Score ≤ 1 TBLC‐based UIP score included 2 patients with IPF, 3 patients with fHP, 2 patients with fNSIP, and 5 patients with unclassifiable ILD. Among these 12 patients, 5 patients received antifibrotic agents, of whom 2 received combination therapy. The period of antifibrotic agent administration for these 2 patients was no earlier than 6 months (Figure 6). As indicated in Figure 4, pulmonary function in most patients showed a stable course regardless of any therapies administered.

Regarding the pathological findings, 9 patients showed prominent inflammatory cells, including 3 with Score ≥ 2 and 6 with Score ≤ 1. Among them, those whose pulmonary function could be assessed during their disease course included 2 patients with Score ≥ 2 and 5 patients with Score ≤ 1. Among the 2 patients with Score ≥ 2, who received an anti‐inflammatory agent only, one had a stable disease course (FVC: 71.6% initially, 92.1% at 8 months later, and 96.3% at 38 months later), and the other one worsened (FVC: 60.5% initially and 46.3% at 13 months later). All 5 patients with Score ≤ 1 had a stable course, with 2 patients receiving no medication (12 and 42 months of follow‐up, respectively), 2 patients receiving only anti‐inflammatory agents (31 and 34 months of follow‐up, respectively), and 1 patient receiving only an antifibrotic agent (FVC: 80.7% initially and 81.1% at 57 months later).

### 3.4 Pathological Evaluation and Adverse Events by TBLC

In most patients, 2 (*n* = 17, 42.5%) or 3 (*N* = 17, 42.5%) pathological samples could be obtained by TBLC (Table S1). Among the specimens determined to be evaluable, 65.0% was classified as grade A and 35.0% as grade B. Definite and probable pathological diagnoses (levels A and B) were made in 42.5% and 55.0%, respectively. Level C indicating poor confidence was assessed in 2.5% of the patients. No serious complications were identified among the patients with adverse events. Bronchial bleeding was identified as mild in 40.0% and as moderate in 30.0% of patients. Moderate pneumothorax was identified in 3 patients (7.5%). No patients required drug therapy or needed oxygen therapy for >24 h as a result of TBLC‐related pneumonia or respiratory failure.

## 4 Discussion

In the present study, nearly half of the patients had a HRCT pattern indeterminate for UIP, indicating that patients who could undergo TBLC were included as clinicians recognized the necessity for determining a treatment strategy in a real‐world setting. This study investigated the usefulness of TBLC when choosing treatment for chronic fibrosing ILD such as idiopathic ILD and fHP. Tomassetti et al. mentioned that both surgical lung biopsy and TBLC can lead to diagnostic reclassification of cases and increased diagnostic confidence, resulting in a significant change in the recommended treatment strategy [22]. TBLC is becoming a valid alternative to surgical lung biopsy, although it does need to be performed in centers with experience, and standardized protocols have the best ratio of risks to diagnostic yield [6]. Taken together, TBLC has shown promise in providing meaningful histological examination when determining a diagnosis by MDD, and early intervention with the appropriate agent can be expected in cases in which determining a treatment strategy is a struggle.

Among patients with PF‐ILD in the INBUILD trial, the annual rate of decline in FVC was significantly lower among the patients who received nintedanib as their antifibrotic agent rather than a placebo [3]. In the RELIEF study, although the quality of evidence was rated as low, pirfenidone as the antifibrotic agent also suppressed the deterioration of FVC in patients with PF‐ILD [23]. Earlier intervention with an antifibrotic agent could lead to more effective prevention of FVC decline [3].

Using changes in pulmonary function during follow‐up as the variable, linear mixed‐effects analysis showed that the declines in %FVC and %DL_CO_ in patients with a TBLC‐based UIP Score ≥ 2 were significantly steeper than those in the patients with a UIP Score ≤ 1. Thus, pathological evaluation using a TBLC‐based UIP Score ≥ 2 may be useful as a predictor of PPF or PF‐ILD in the future. Clinicians should keep this possibility in mind in the early phase of treating each patient. In practice, in the groups with antifibrotic agent use, many patients with a TBLC‐based UIP Score ≥ 2 received an antifibrotic agent within 6 months after the diagnosis by MDD. Moreover, in the groups receiving combination therapy, most patients were also administered an antifibrotic agent within 6 months of the diagnosis by MDD. Thus, if we could intervene more quickly by administering an antifibrotic agent early to patients with worsened pulmonary function, the patients with PF‐ILD or PPF in the present study who underwent TBLC might have benefitted from early introduction of an antifibrotic agent. Expansion of fibrotic change in patients with both a radiological and pathological UIP pattern can lead to a high risk of PPF or PF‐ILD, which means a poorer prognosis for any of the ILDs [5, 24, 25]. Therefore, the descriptive research undertaken in the present study suggested that additional pathological information obtained from TBLC can contribute to early medical intervention particular in terms of antifibrotic agent administration in clinical practice, although a comparative study to assess this might be difficult.

As cautionary points, some patients with a TBLC‐based UIP Score ≥ 2 had a stable course, whereas others with a UIP Score ≤ 1 had a progressive course. Clearly, not only pathological findings but also clinicoradiological information is important. By performing MDD in the assessment of ILD in real life, we want to highlight the importance of early multidisciplinary assessment, which improves the therapeutic decision‐making strategy [15, 16, 26, 27]. In addition, as TBLC specimens can be inadequate, clinicians should not place too much emphasis on pathological findings based on this method in the therapeutic assessment of ILD [10].

More patients with a TBLC‐based UIP Score ≤ 1 than Score ≥ 2 showed a high frequency of prominent inflammatory cells in their pathological findings, which indicates that this finding may be associated with a better response to anti‐inflammatory agents [10, 28]. In the present study, pulmonary function in 2 of the 3 patients who received an anti‐inflammatory agent improved and then stabilized for more than 2.5 years. Our previous study of CTD‐ILD found that TBLC may be useful when it is difficult to judge which treatment should be prioritized, that with anti‐inflammatory or antifibrotic agents, even if the patient shows a UIP pattern [11]. The present study also suggested the importance of this point. However, one of the present patients had a refractory response to the anti‐inflammatory agent. Thus, clinicians should not be overconfident when treating based only on pathological findings; MDD is obviously still important.

The present study has several limitations. First, the number of patients in this retrospective study was small, and there is selection bias because only those patients who could undergo TBLC were included as a clinician determined the necessity for the procedure and enrollment into the study. Second, clear identification of a statistically significant group was difficult. Generally, descriptive research cannot be used to establish cause and effect relationships. Although this study suggests that those with more UIP‐like features on pathology have a greater decline in lung function, this is not necessarily automatically predictive of PPF/PF‐ILD. However, we speculate that judgment of whether a patient has UIP‐like lesions radio‐pathologically is meaningful when determining a strategy for treatment because clinicians should consider the possibility that patients will develop PPF/PF‐ILD over the long run if they have UIP‐like lesions when determining a treatment strategy [5, 10, 21]. Indeed, descriptive research including therapeutic assessment and disease behavior based on changes in pulmonary function was done, which may present beneficial information to clinicians. Third, the present analysis combined idiopathic ILD and fHP into one entity. In fHP, appropriate antigen avoidance is the most important therapy rather than that by anti‐inflammatory and antifibrotic agents [29, 30]. In most of the patients with fHP in this study, we could not identify the obvious antigen, which might greatly influence each patient's disease course.

In conclusion, the present study showed that in patients who underwent TBLC, a TBLC‐based UIP Score ≥ 2 indicated the increased possibility of a progressive fibrosis course that may prove helpful in predicting PPF/PF‐ILD, which may benefit from early introduction of antifibrotic agent by treating clinicians. Additional or prospective studies with a larger sample size will be necessary to further identify therapeutic decision‐making strategies in ILD.

## Author Contributions

M.T. and H.Y. acquired the data; M.T., H.Y., T.T., S.S., H.O., K.K., T.O., R.K., K.A., H.S., M.A., J.A., and H.M. analyzed and interpreted the clinical data; and M.T., H.Y., and H.M. drafted the manuscript. All authors read and approved the final manuscript.

## Ethics Statement

This study was approved by the Ethics Committee of Medical Research, Saitama Red Cross Hospital (No. 19‐C) and was conducted in accordance with the Declaration of Helsinki. The requirement for written informed consent was waived by the Ethics Committee of Medical Research based on the retrospective nature of this study.

## Conflicts of Interest

H.Y. and S.S. received honoraria from Nippon Boehringer Ingelheim Co. Ltd. The other authors declare no conflicts of interest.

## Supporting information


**Table S1** Pathological evaluation and adverse events caused by TBLC.

## Data Availability

The datasets used and/or analyzed during the current study are available from the corresponding author on reasonable request.
