# Supplementary material for: Usefulness of Transbronchial Lung Cryobiopsy When Starting Antifibrotic Treatment and Predicting Progressive Fibrosing Interstitial Lung Disease: Descriptive Research
Source: Clin Respir J. 2024 Jul 16;18(7):e13809. doi: 10.1111/crj.13809 (PMC11251805; doi:10.1111/crj.13809)
Supplement: Supplementary file 1 — Table S1 Pathological evaluation and adverse events caused by TBLC. [file CRJ-18-e13809-s001.docx]

| **Table S1**  Pathological evaluation and adverse events caused by TBLC. | |
| --- | --- |
| Total | No. of patients, n = 40 |
|  | n (%) |
| Number of samples | |
| 1 | 5 (12.5) |
| 2 | 17 (42.5) |
| 3 | 17 (42.5) |
| 4 | 1 (2.5) |
| Pathological quality and quantity |  |
| Quality score |  |
| Grade A | 26 (65.0) |
| Grade B | 14 (35.0) |
| Grade C | 0 (0.0) |
| Pathological confidence | |
| Level A | 17 (42.5) |
| Level B | 22 (55.0) |
| Level C | 1 (2.5) |
| Adverse events |  |
| Bronchial bleeding | |
| Mild | 16 (40.0) |
| Moderate | 12 (30.0) |
| Pneumothorax |  |
| Mild | 2 (5.0) |
| Moderate | 3 (7.5) |
| TBLC, transbronchial lung cryobiopsy. | |
